# Oxaliplatin and Its Enantiomer Induce Different Condensation Dynamics of Single DNA Molecules

**DOI:** 10.1371/journal.pone.0071556

**Published:** 2013-08-12

**Authors:** Hong-Yan Zhang, Yu-Ru Liu, Chao Ji, Wei Li, Shuo-Xing Dou, Ping Xie, Wei-Chi Wang, Ling-Yun Zhang, Peng-Ye Wang

**Affiliations:** Key Laboratory of Soft Matter Physics, Beijing National Laboratory for Condensed Matter Physics, Institute of Physics, Chinese Academy of Sciences, Beijing, China; Max Planck Institute for Polymer Research, Germany

## Abstract

The interactions of DNA with oxaliplatin (Pt(R,R-DACH)) or its enantiomer (Pt(S,S-DACH)) were investigated using magnetic tweezers and atomic force microscope. In the process of DNA condensation induced by Pt-DACH, only diadducts and micro-loops are formed at low Pt-DACH concentrations, while at high Pt-DACH concentrations, besides the diadducts and micro-loops, long-range cross-links are also formed. The diadduct formation rate of Pt(R,R-DACH) is higher than that of Pt(S,S-DACH). However, the proportions of micro-loops and long-range cross-links for Pt(S,S-DACH) are higher than those for Pt(R,R-DACH). We propose a model to explain these differences between the effect of Pt(R,R-DACH) and that of Pt(S,S-DACH) on DNA condensation. The study has strong implications for the understanding of the effect of chirality on the interaction between Pt-DACH and DNA and the kinetics of DNA condensation induced by platinum complexes.

## Introduction

The clinical success of cisplatin as an anticancer drug has resulted in the synthesis of new platinum complexes as potential drug candidates. Among these complexes, oxaliplatin has shown a broad antitumor activity [1]–[4] and a lack of cross-resistance with cisplatin [3], [5], [6]. Thus, oxaliplatin has obtained worldwide approval for the clinical treatment of metastatic colorectal carcinoma as a third-generation platinum complex [7].

The antitumor effect of platinum complexes mainly results from their ability to damage DNA by forming various types of covalent adducts, which affect essential processes such as replication and transcription and thus lead to cell death [3], [8]–[10]. Once inside the body, the platinum complexes undergo ligand substitution with the help of chloride, phosphate, and carboxylate to form positively charged monoaquated and diaquated species [11]. Previous studies have shown that only these aquated forms bind to DNA [12], [13]. The binding process consists of two steps. In the first step, positively charged aquated platinum complexes approach DNA quickly with electrostatic attraction and preferentially coordinate to the N7 atom of guanine residues to form monoadducts. The second binding step takes place at adjacent purine residues and the diadducts are formed [3], [14]. The kinds and proportions of adducts of oxaliplatin are very similar to those of cisplatin [15], [16]. The 1,2-GG/AG diadduct has generally been recognized as an important adduct for anticancer activity because of its high proportion and relatively remarkable conformational alterations induced in DNA.

**Figure 1:**
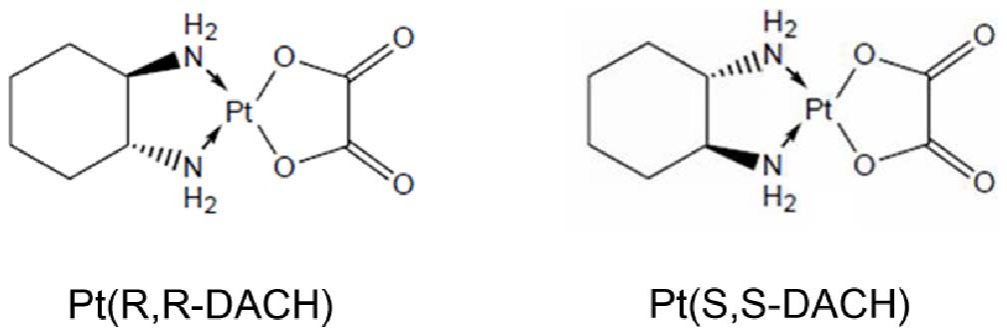
Chemical structures of Pt(R,R-DACH) and Pt(S,S-DACH).

Oxaliplatin has gained increasing attention because of its intrinsic chirality. The trans form of Pt-DACH can exist as trans-1R,2R (Pt(R,R-DACH), oxaliplatin, RR for short) and trans-1S,2S (Pt(S,S-DACH), SS for short) isomers (Figure 1). When the two enantiomers interact with a double-helical DNA, which also has a chiral structure, there should be some differences. Indeed, differences in antitumor activity were observed although it is now accepted that these were probably not significant [17]–[21]. Obvious differences in mutagenicity were also observed [22], [23]. Usually, the more active enantiomer was the less mutagenic one. This interesting observation inspired the enthusiasm to identify the causes of these differences. Previous studies have shown that there were subtle differences between the sites and types of adducts [24], the NMR spectra of 1,2-GG adduct [25], the electronic structure [26], and the molecular model of 1,2-GG adduct [27] of RR and those of SS [28]. Another study of the 1,2-GG adducts in the TGGT sequence demonstrated that the chirality of Pt-DACH affected their biological effects [13]. In addition to the analyses of the Pt-DACH adducts, Reedijk [29] has pointed out that the kinetics of DNA binding is crucial to all properties related to antitumor activity. Therefore, our present work mainly focused on the DNA binding kinetics of Pt-DACH.

Single molecule techniques are helpful methods to study the interactions between DNA and multivalent cations [30], [31], proteins [32]–[34], as well as platinum drugs [35]–[39]. Hou et al. [35] studied the interaction between DNA and cisplatin by magnetic tweezers and atomic force microscopy (AFM) and proposed a model to explain the mechanism of DNA condensation induced by cisplatin. They proved that the model was also applicable to DNA condensation induced by oxaliplatin.

In this paper, we employed magnetic tweezers and AFM to study the interaction between DNA and Pt-DACH. The differences between the effect of Pt(R,R-DACH) and that of Pt(S,S-DACH) on DNA condensation were found.

## Results and Discussion

DNA condensation induced by platinum complexes usually involves three main kinds of adducts, namely, diadducts, micro-loops [40], and long-range cross-links [35]. Diadducts are mainly involved in 1,2-GG or 1,2-AG intrastrand crosslinks, which are characterized as local DNA bending in AFM images (inset in Figure below). Their effect on DNA condensation is confined to several bases. A micro-loop is a DNA loop induced by the binding of a monoadduct to a distant base, which has a higher and larger protuberance than the size of a normal DNA in AFM images (inset in Figure below) [35]. We estimate that there are approximately 150 base pairs between the two binding sites of a micro-loop from the AFM images (see below). Thus, the effect of a micro-loop involves several hundred bases. If only these two kinds of adducts are formed, it would take a long time to form highly condensed DNA molecules. Thus, the long-range cross-links between DNA segments at certain distances or micro-loops have an important function in DNA condensation.

### Effects of Pt-DACH on DNA Elasticity

**Figure 2:**
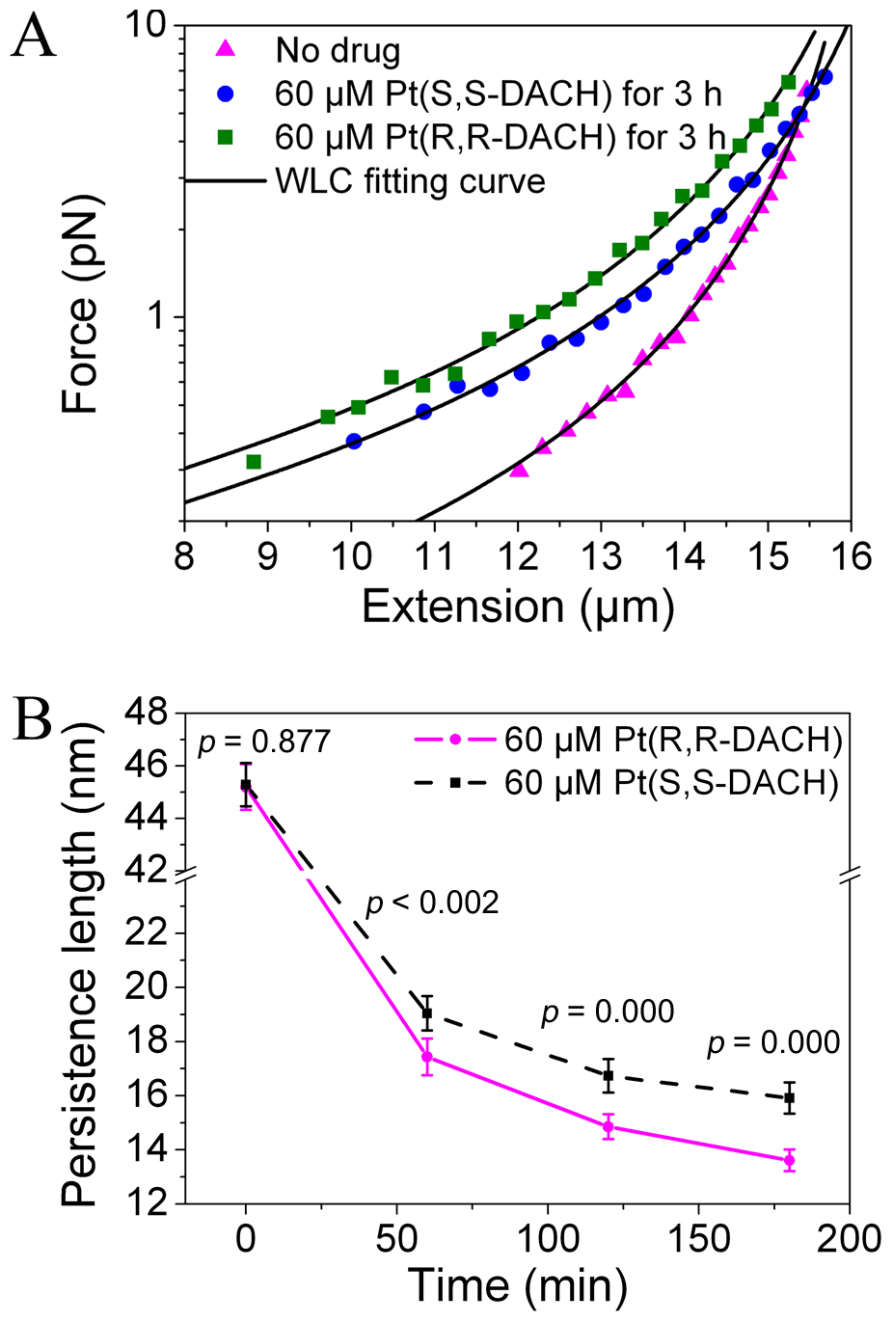
Force-extension curves and persistence length as a function of incubation time. (A) The force-extension curves of λ-DNA without drug (*L* = 16.5±0.04 µm, *P* = 44.0±2.1 nm), with 60 µM Pt(S,S-DACH) incubated for 3 h (*L* = 17.3±0.08 µm, *P* = 16.5±1.0 nm ) and 60 µM Pt(R,R-DACH) incubated for 3 h (*L* = 17.1±0.09 µm, *P* = 12.9±0.7 nm ). The data were fitted by the WLC model (Eq. 1). (B) The persistence lengths of λ-DNA treated with 60 µM Pt(R,R-DACH) or Pt(S,S-DACH) for different incubation times. Each data point was the mean of twenty independent measurements. The error bars corresponded to 95% confidence intervals. The difference was considered statistically significant when the *p* value of two-sample t-test was less than 0.05.

The force-extension curve of single DNA molecule either without drug or with 3-h incubation of 60 µM Pt-DACH was measured (Figure 2A) to test whether Pt-DACH affected the elasticity of DNA and whether the WLC model was applicable to DNA treated with Pt-DACH of low concentration. Pt-DACH changed the elasticity of DNA and the force-extension curve of the DNA treated with Pt-DACH can be fitted well by the WLC model.

A series of force-extension curves were measured to further study the differences between RR and SS in terms of DNA elasticity. The persistence length as a function of incubation time was obtained by fitting the data with the WLC model (Figure 2B). The persistence length at *t* = 0 min represents the result obtained before adding Pt-DACH. In the case of RR treatment, the persistence length is reduced from 45.2±0.9 nm (mean ±95% confidence interval) to 17.4±0.7 nm with 60-min incubation, and is further reduced to 13.6±0.4 nm with 180-min incubation. In the case of SS treatment, the persistence length is reduced from 45.3±0.8 nm to 19.0±0.6 nm with 60-min incubation, and is further reduced to 15.9±0.6 nm with 180-min incubation. The change in persistence length induced by RR is larger than that induced by SS. Similar results are obtained for 30 µM Pt-DACH (data not shown). The difference, albeit small, is important for the interaction between DNA and Pt-DACH. The two-sample t-test is used to analyze the difference in persistence length between RR and SS. The difference is considered statistically significant when the *p* value is less than 0.05. *P* values at 60, 120 and 180 min are all less than 0.05, which mean that the difference between RR and SS is significant.

The major 1,2-GG or 1,2-AG intrastrand crosslinks formed by Pt-DACH bend the helix axis toward the major groove and locally unwind DNA [41], [42], disturbing the base-stacking interactions. The electrostatic attraction between aquated Pt-DACH and phosphate groups on DNA reduces the phosphate-phosphate charge repulsion. The combined effect of the damage to base-stacking interactions and the reduction of the phosphate-phosphate charge repulsion induce instability and more flexibility to the DNA. Thus, the persistence length is reduced. Yan et al. [43] explained the reduction of DNA persistence length induced by local bending through the viewpoint of entropic elastic stiffness, which is also applicable to our results.

We infer that the differences between RR and SS in terms of DNA elasticity result either from their differences in diadduct formation rate or from their differences in the bending and unwinding degree. The AFM experiments on bending and unwinding degree show no obvious difference between them (see bending angle distribution). As a result, we infer that the diadduct formation rate of RR is higher than that of SS and, thus, the change in persistence length induced by RR is larger than that induced by SS. This finding, which is consistent with the result of Boudny et al. [44], indicates that SS exhibits a slower formation kinetics of diadduts than RR.

### Dynamics of DNA Condensation Induced by Pt-DACH

**Figure 3:**
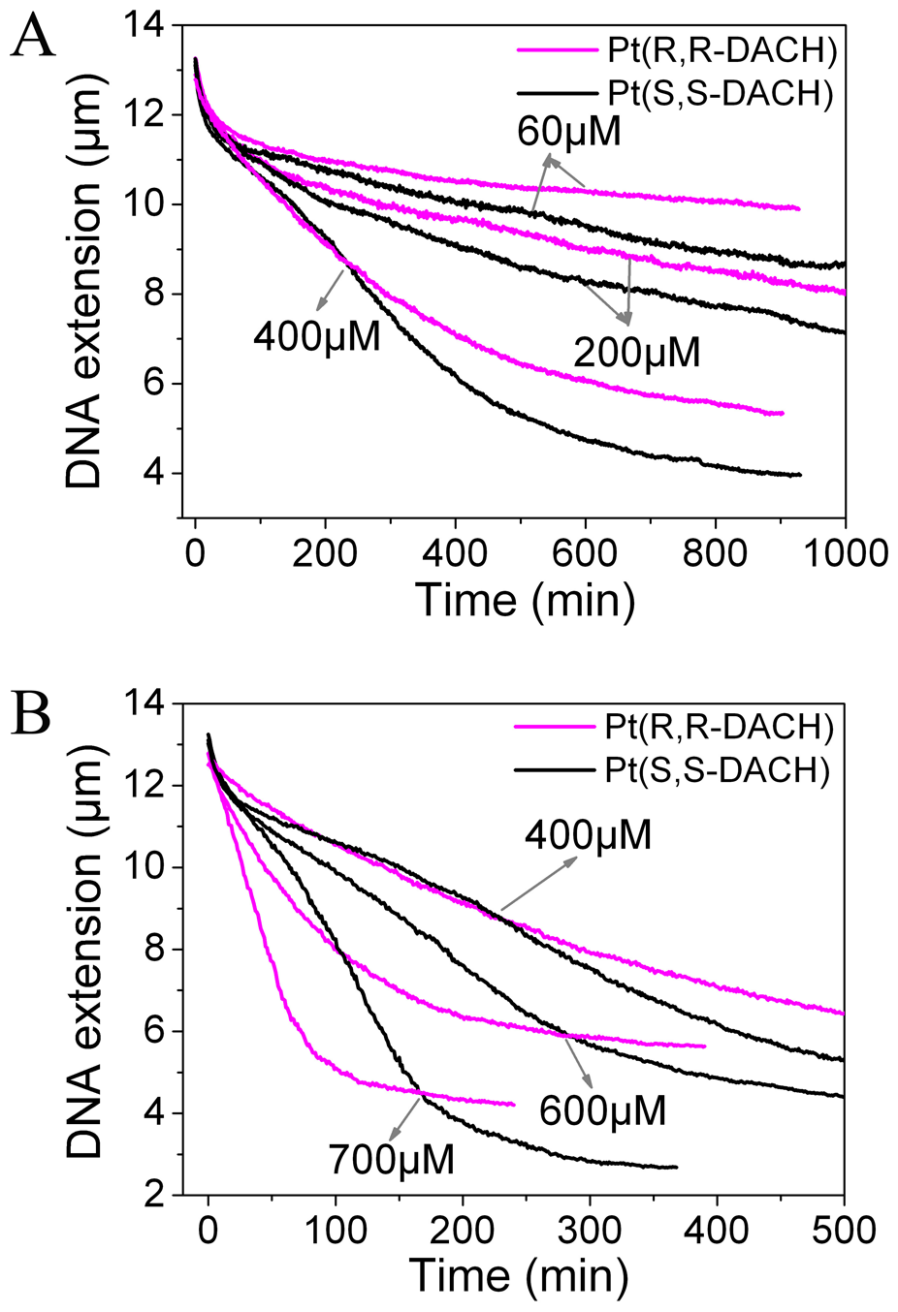
DNA extension versus time of λ-DNA treated with Pt(R,R-DACH) or Pt(S,S-DACH). Each data curve was the average of at least three independent measurements.

**Figure 4:**
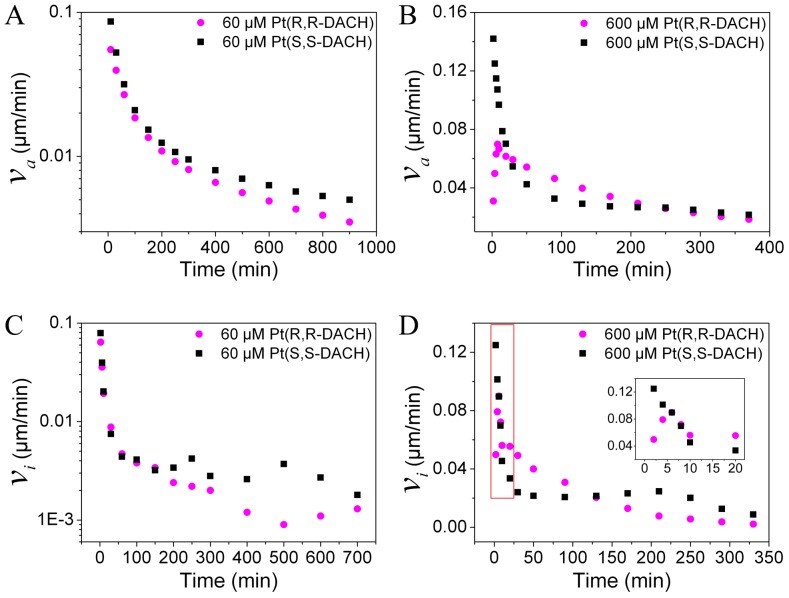
The average DNA condensation rates (*ν_a_*) and the instantaneous DNA condensation rates (*ν_i_*).

The extensions of DNA incubated with Pt-DACH at different concentrations were measured with time by magnetic tweezers (Figure 3). Since the reaction between DNA and Pt-DACH was very slow due to the bulk DACH ligand of Pt-DACH, high concentrations of Pt-DACH were used here to accelerate the reaction. The stretching force was approximately 0.8 pN. At any concentration, the final DNA condensation degree induced by SS is always higher than that induced by RR. The shapes of the curves at concentrations below 400 µM are obviously different from those at concentrations above 400 µM. We calculate both the average DNA condensation rates and the instantaneous DNA condensation rates to further study this phenomenon. Two typical results are shown in Figure 4. When the concentrations are below 400 µM, the average DNA condensation rates for RR are initially similar to those for SS and then become slower than for SS. When the concentrations are above 400 µM, the rates for RR are initially slower, then become faster, and ultimately become slower than those for SS. The same behaviors are obtained for the instantaneous DNA condensation rates.

**Figure 5:**
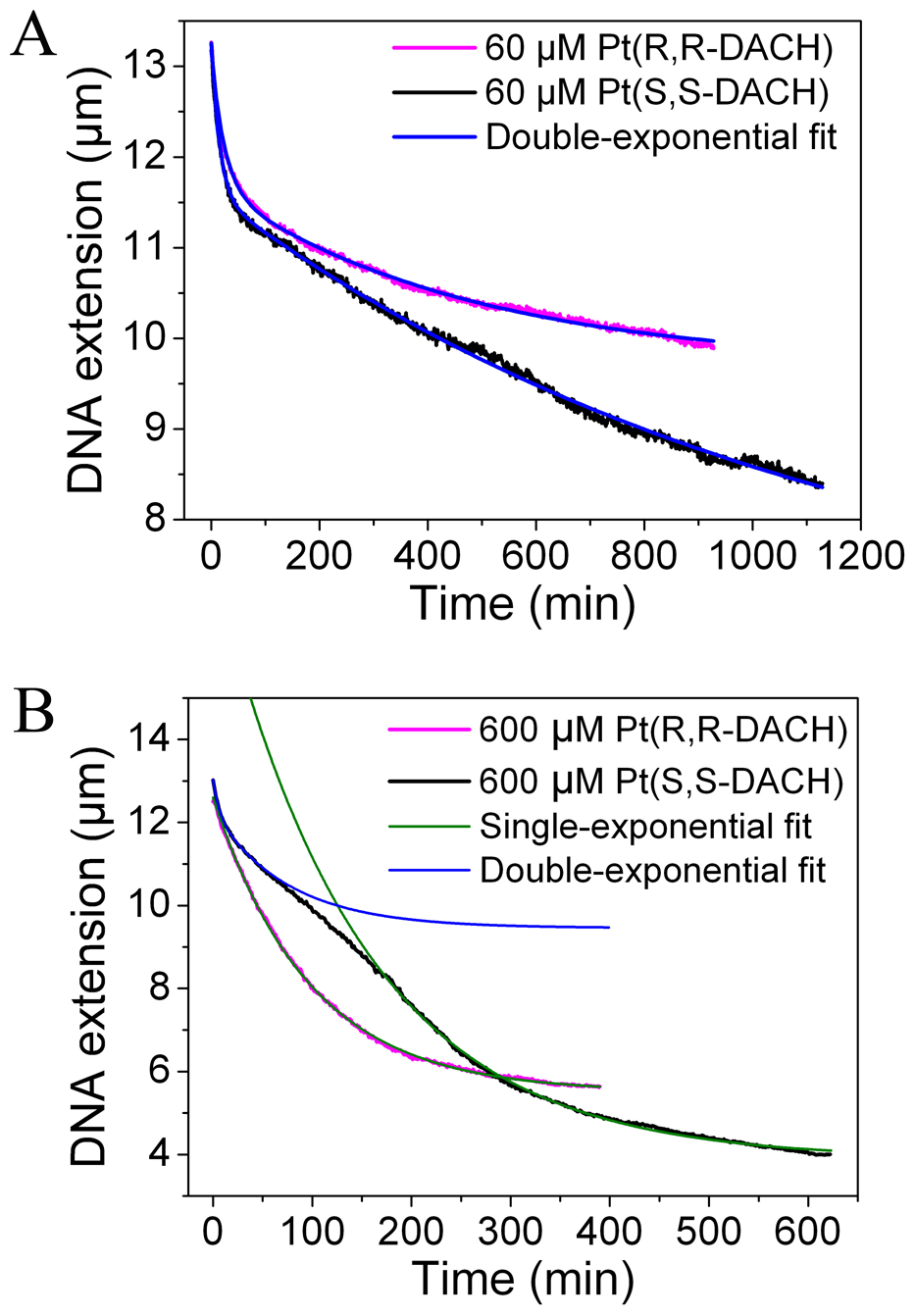
Fittings of the DNA condensation data using exponential decay models. (A) Double-exponential curve fitting of the data for both Pt(R,R-DACH) (*T*_1_ = 23.89 min, *T*_2_ = 473.27 min) and Pt(S,S-DACH) (*T*_1_ = 14.74 min, *T*_2_ = 1130.61 min) at 60 µM. (B) Single-exponential curve fitting of the data for Pt(R,R-DACH) (*T* = 97.13 min) and the last part of Pt(S,S-DACH) (*T* = 146.98 min) at 600 µM. The initial part of the data for Pt(S,S-DACH) was fitted by a double-exponential decay model (*T*_1_ = 6.07 min, *T*_2_ = 76.23 min).

To explain the above phenomena, the experimental data are firstly fitted using exponential decay models to analyze the reactions in the process of DNA condensation. Both the RR and SS data can be fitted well by a double-exponential decay model for concentrations below 400 µM (Figure 5A), which are assumed to correspond to two concurrent pseudo-first order reactions. As can be seen from AFM experiments (see effects of Pt-DACH on DNA structure), the two reactions correspond to the formations of diadducts and micro-loops. Compared with diadducts and micro-loops, monoadducts cannot induce intrinsic bending [45], [46] and thus they have negligible effect on DNA extension in our experiments. For concentrations above 400 µM (Figure 5B), the initial part of the SS data can be fitted by a double-exponential decay model, which is similar to that for concentrations below 400 µM. The last part of the SS data can be fitted by a single-exponential decay model, which is assumed to correspond to a pseudo-first order reaction. As can be seen from AFM experiments (see effects of Pt-DACH on DNA structure), this reaction corresponds to the formation of long-range cross-links. Although the RR data can be fitted by a single-exponential decay model, obviously more than one reaction occurs, as inferred from the discontinuous change in the slope of the instantaneous rate versus time curve (Figure 4D).

Based on the fitting results of the curves in Figure 5A, the formation rates of diadducts and micro-loops are obtained through the derivative calculations. The formation rate of diadducts is higher than that of micro-loops obtained from AFM images (see effects of Pt-DACH on DNA structure). Thus, the faster exponential decay corresponds to diadducts and the slower to micro-loops. The diadduct formation rate of RR is higher than that of SS and the formation rate of micro-loops for RR is lower than that of SS, although the diadduct formation rate of RR is lower than that of SS in the first few minutes for 60 µM Pt-DACH, which is probably due to the loss of some data because the diadducts are formed for RR during the injection of Pt-DACH (approximately 3 min). The diadduct formation rate decreases rapidly over time, whereas the formation rate of micro-loops decreases relatively slowly.

The differences between RR and SS on DNA condensation will be discussed in detail in the next section (see proposed model). Here, we only briefly discuss the differences as follows. Because of the higher diadduct formation rate of RR compared with that of SS, there are more opportunities to form micro-loops or long-range cross-links for SS than for RR. For concentrations below 400 µM, there are two reactions: the formations of diadducts and micro-loops. Although the diadduct formation rate of RR is larger than that of SS, the average DNA condensation rate for SS is higher than that for RR in the last phase of DNA condensation because of the greater contribution of micro-loops to DNA condensation and the higher formation rate of micro-loops for SS. For concentrations above 400 µM, besides the two reactions mentioned above, there is another reaction: the formation of long-range cross-links. However, its starting time lags for SS. In the initial phase of DNA condensation, the diadduct formation rate of RR is much higher than that of SS, which leads to the higher average condensation rate and instantaneous condensation rate for RR compared with those for SS (long-range cross-links also play a key role, which is proved by AFM experiments (see effects of Pt-DACH on DNA structure)). In the last phase, the instantaneous condensation rate for SS is faster than that for RR because of the occurrence of long-range cross-links. DNA condensation degree induced by SS is higher than that induced by RR because of the greater contribution of micro-loops and long-range cross-links to DNA condensation and the higher probability to form micro-loops and long-range cross-links for SS. The record time may be not long enough to observe the formation of long-range cross-links for Pt-DACH at low concentrations.

The first requirement to form long-range cross-link is the formation of monoadduct. The second requirement is that one base, which is separated from the monoadduct by several hundred bases in DNA, is close to the monoadduct. Thus, a large amount of monoadducts, a lower persistence length of DNA, and a higher DNA condensation degree can facilitate the formation of long-range cross-link. The higher formation rate of diadduct for RR results in the lower persistence length and the higher DNA condensation degree compared with those for SS. Thus, the start time of long-range cross-link for RR is earlier than that of SS. The lower diadduct formation rate for SS results in more monoadducts than for RR. Thus, the proportion of long-range cross-links is higher for SS than for RR.

It is important to note that we cannot directly prove the formations of diadducts, micro-loops and long-range cross-links through experiments by using magnetic tweezers. The above analyses of the processes of DNA condensation induced by Pt-DACH are based on the hypothesis that the process of DNA condensation in the magnetic tweezers experiment is similar to that in the AFM experiment. Under this premise, combining the magnetic tweezers experiments with AFM experiments, we can have a relatively deep understanding of the process of DNA condensation induced by Pt-DACH.

### Effects of Pt-DACH on DNA Structure

We used AFM to scan DNA incubated with RR or SS to directly observe the effect of Pt-DACH on DNA structure.

**Figure 6:**
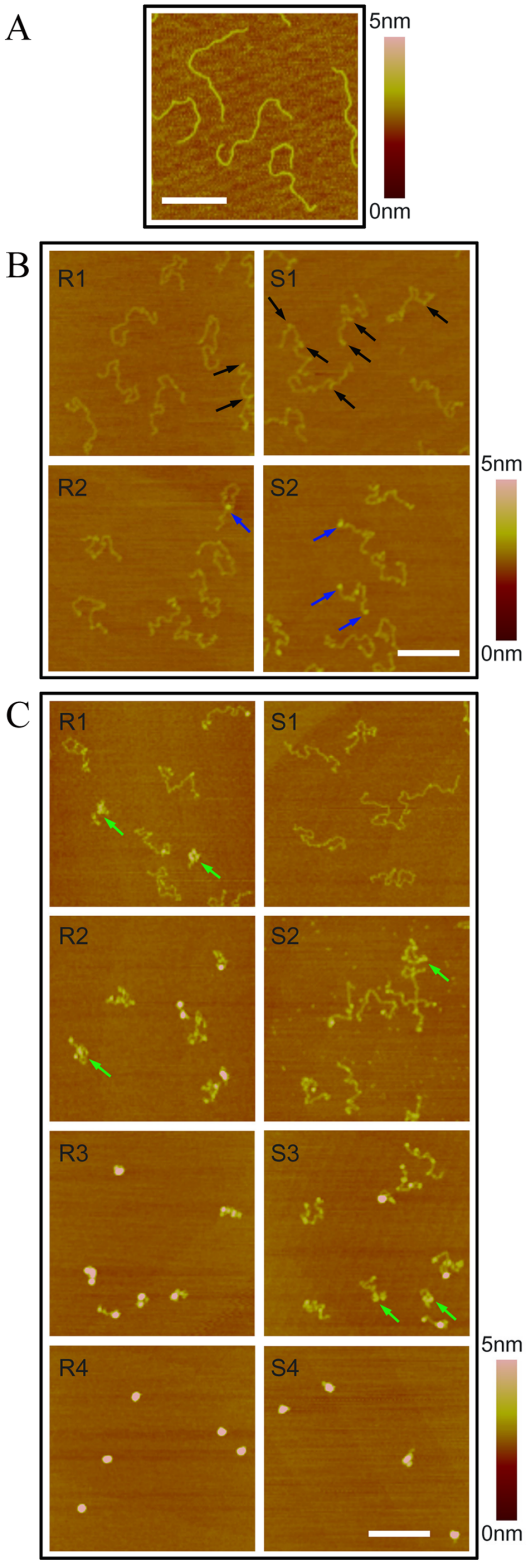
AFM images of 2-kb DNA incubated with Pt-DACH. (A) In the absence of Pt-DACH treatment. (B) The concentration was 60 µM for both Pt(R,R-DACH) and Pt(S,S-DACH). The molar ratio of Pt-DACH complex to nucleotide, *r_i_*, was about 3. “R” and “S” represent Pt(R,R-DACH) and Pt(S,S-DACH), respectively. “1″ and “2″ represent incubation times of 2 h and 24 h, respectively. The micro-loop was marked by the black arrow. The large-sized micro-loop was marked by the blue arrow. (C) The concentration was 600 µM for both Pt(R,R-DACH) and Pt(S,S-DACH). *r_i_* was about 30. “1″, “2″, “3″ and “4″ represent incubation times of 1 h, 2 h, 4 h and 8 h, respectively. The long-range cross-link was marked by the green arrow. All images were of the same size, and the scale bar was 300 nm.

The AFM image of DNA molecules in the absence of RR or SS treatment is shown in Figure 6A. The DNA molecules are in the extended state. The curvature along the entire DNA molecule changes continuously except for a few local sharp bending angles which may be caused by the nick of the DNA molecule. Because the number of local bending angles in normal DNA molecule is very low comparing with that incubated with Pt-DACH, we think all of the local bending angles are induced by Pt-DACH approximately.

The AFM images of DNA molecules incubated with 60 µM RR or SS are shown in Figure 6B. After 2-h incubation, local DNA bending, which is mainly the result of the formation of diadducts, and micro-loops (marked by the black arrow, with a height of about 0.6 nm) are observed for both RR (Figure 6B R1) and SS (Figure 6B S1). The proportion of local DNA bending is greater than that of micro-loop for both RR and SS, which indicates that the formation rate of diadduct is higher than that of micro-loop (data not shown). The proportion of micro-loops for SS is greater than that for RR (data given below). After 24-h incubation, there are essentially still only two kinds of adducts: diadducts and micro-loops. The proportion of micro-loops for SS (Figure 6B S2) is still greater than that for RR (Figure 6B R2). Unlike the micro-loops that formed after 2-h incubation, some large-sized loops with a height of approximately 1.2 nm (marked by the blue arrow) are formed. These large-sized loops are classified as micro-loops compared with long-range cross-links whose effect on DNA condensation degree is very large. In conclusion, two kinds of adducts, namely, diadducts and micro-loops, are formed in the process of DNA condensation induced by 60 µM RR or SS. The proportion of micro-loops is higher for SS than for RR.

The AFM images of DNA molecules incubated with 600 µM RR or SS are shown in Figure 6C. After 1-h incubation, local DNA bending, micro-loops, and long-range cross-links (marked by the green arrow) are formed for RR (Figure 6C R1), whereas only local DNA bending and micro-loops are formed for SS (Figure 6C S1). The DNA condensation degree for RR is higher than that for SS (data given below). After 2-h incubation, highly condensed DNA molecules are observed for RR (Figure 6C R2). Meanwhile, long-range cross-links are observed for SS (Figure 6C S2). After 8-h incubation, all DNA molecules incubated with RR (Figure 6C R4) are condensed into compact hemispherical structures with a height of about 6 nm, whereas the condensation process is not yet complete for SS (Figure 6C S4). In conclusion, three kinds of adducts, namely, diadducts, micro-loops, and long-range cross-links, are formed in the process of DNA condensation induced by 600 µM RR or SS. The start time of long-range cross-links for SS is later than that for RR.

In the following, we will present statistical analyses of the stretching distance, the bending angle, the number of micro-loops formed in 100 DNA molecules incubated with Pt-DACH at 60 µM to describe the DNA structural differences between RR and SS. For 600 µM, because the reactions are too rapid only the stretching distance is analyzed here. The volume of the completely condensed DNA molecule induced by 600 µM Pt-DACH is also statistically analyzed to measure DNA condensation degree.

### Statistics of DNA Stretching Distance

**Figure 7:**
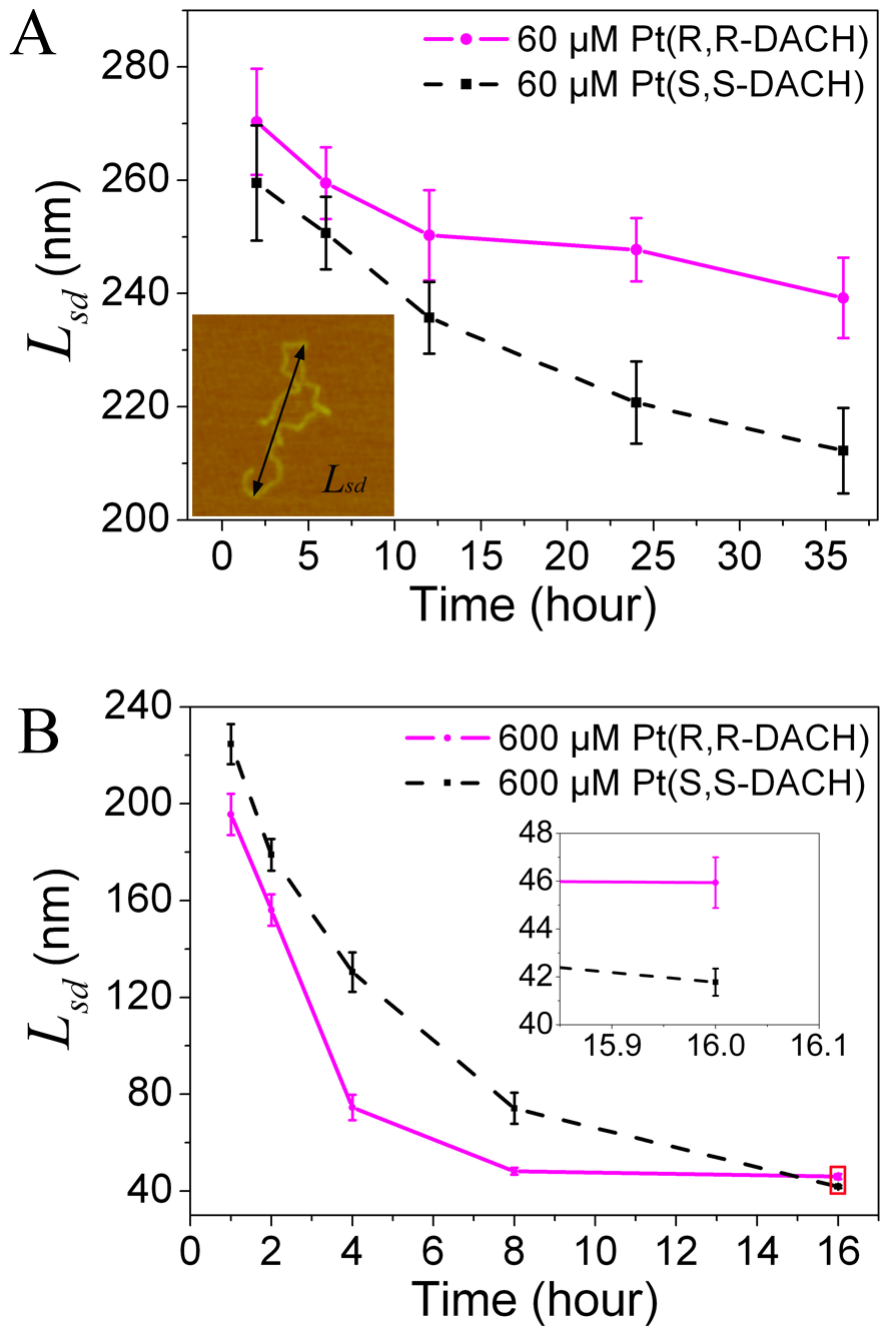
Average stretching distance (*L_sd_*) of 2-kb DNA incubated with Pt-DACH. (A) The concentration was 60 µM for both Pt(R,R-DACH) and Pt(S,S-DACH). (B) The concentration was 600 µM for both Pt(R,R-DACH) and Pt(S,S-DACH). All data were the mean of approximately 150 DNA molecules. The error bars corresponded to 95% confidence intervals. Inset: an illustration of the definition of stretching distance.

The end-to-end distance is generally used to describe the statistical behavior of a polymer chain, such as a linear DNA. However, the end-to-end distance is not suitable to describe the DNA in the presence of platinum drugs because of partial DNA bending and unwinding. Thus, the concept of stretching distance is put forward in this study to characterize the degree of DNA condensation. The stretching distance of DNA refers to the longest distance between two points of a single DNA molecule (inset in Figure 7A), which is similar to the definition of long-axis length by Yoshikawa et al. [47].

The statistical results of the average stretching distances at different times are shown in Figure 7. The average stretching distance for 60 µM RR is almost the same as that for 60 µM SS initially, and then becomes longer than that for SS (Figure 7A). The average stretching distance for 600 µM RR is shorter than that for 600 µM SS initially, and then becomes longer than that for SS (Figure 7B). These results are consistent with those obtained from experiments by using magnetic tweezers (Figure 5), which could be considered as a proof on the hypothesis that the process of DNA condensation in the magnetic tweezers experiment is similar to that in the AFM experiment.

### Bending Angle Distribution

Normal DNA molecules have difficulty in forming sharp bending angles and the curvature along the entire DNA molecule changes continuously because of its rigidity. However, the intrastrand crosslinks of platinum complexes bend the DNA helix axis toward the major groove and locally unwind the DNA. The NMR solution structure and the crystal structure of the 1,2-GG intrastrand crosslink of oxaliplatin demonstrated that 1,2-GG crosslink bended the double helix by approximately 30° toward the major groove [41], [42]. A novel feature of this crystal structure was the presence of a hydrogen bond between the pseudoequatorial NH hydrogen atom of the RR ligand and the O6 atom of the 3′-G of the platinated d(GG) site. This provides structural evidence for the importance of chirality in mediating the interaction between oxaliplatin and double-stranded DNA [41]. However, this feature was not observed via NMR [42]. Thus, it is necessary to determine if the chiral carbon atom of the DACH carrier ligand in the intrastrand adduct affects DNA conformation such as bending and unwinding.

**Figure 8:**
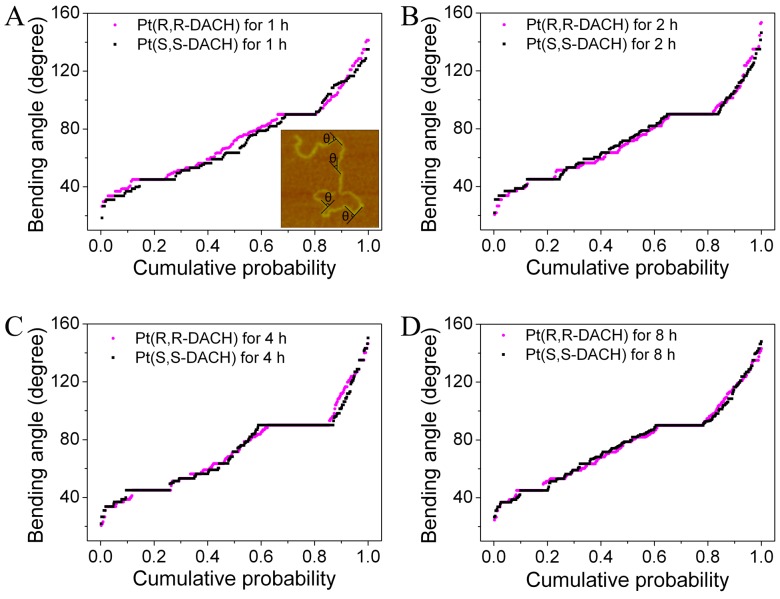
Cumulative probability of the bending angle of DNA induced by 60 µM Pt(R,R-DACH) or Pt(S,S-DACH). The incubation times were 1 h (A), 2 h (B), 4 h (C) and 8 h (D). Approximately 300 angles were measured for each time point. Inset: an illustration of the definition of the bending angle (θ).

The bending angles are statistically analyzed for both RR and SS at different incubation times (Figure 8), where only the sharp bending angle, which is easy to identify, is measured (inset in Figure 8A). The bending angle can be induced by more than one Pt-DACH molecule. The local unwinding of DNA induced by Pt-DACH can affect the bending angle of DNA on the mica surface, although the weak adsorption between DNA and mica mediated by magnesium ions has little influence on the DNA structure. In spite of these unsatisfactory problems, the statistical result of the bending angle as an average can still explain the result obtained from the experiment by using magnetic tweezers, which is also the average effect of lots of Pt-DACH molecules on DNA. As shown in Figure 8, the difference between RR and SS in terms of bending angle is small at the same incubation time, which indicates that the average effect of RR on bending angle is almost the same as that of SS. The findings of previous studies on both the electronic structures of the two enantiomers and the NMR spectra of Pt(R,R-DACH)(d(GpG)) and Pt(S,S-DACH)(d(GpG)) are consistent with our results [25], [26]. A common feature of all incubation times is the extent of the bending angle which spans a wide range from 20° to 160° and the majority is between 45° and 90°. Specially, the bending angles of 45° and 90° have high proportions. DNA bending and unwinding induced by the 1,2-GG intrastrand crosslink of RR or SS in the sequences TGGT, CGGA, and AGGC were studied previously using the gel electrophoresis retardation assay [13]. The authors believed that the DNA bending and unwinding were similar for both enantiomers in the CGGA and AGGC sequences. However, several differences in the TGGT sequence were observed. Interestingly, the sum of the bending angle and the unwinding angle measured via electrophoretic retardation is approximately 48°, which is very close to the value (45°) obtained by our statistical analyses of DNA bending angles on the mica surface. We infer that the bending angle of DNA on 2D surface may be influenced by the combined effect of the bending and unwinding of DNA in 3D space. In conclusion, no difference between RR and SS in terms of the bending angle of DNA is observed.

### Numbers of Micro-loops per 100 DNA Molecules

**Figure 9:**
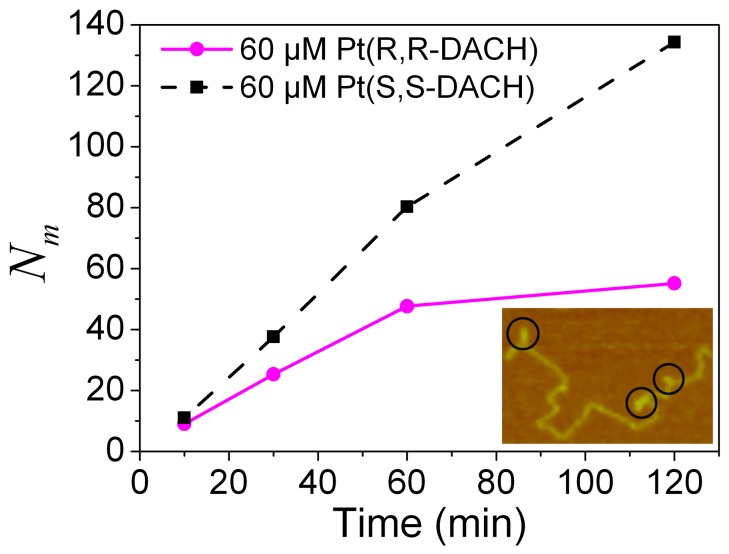
Numbers of micro-loops per 100 DNA molecules (*N_m_*). Approximately 150 DNA molecules were analyzed for each data point. Inset: an illustration of the definition of micro-loop.

The probabilities of micro-loops are statistically analyzed for both RR and SS, and the numbers of micro-loops per 100 DNA molecules at different incubation times are shown in Figure 9. At 10 min, the numbers are small for both RR and SS, and the difference between the two cases is also small. However, at 60 min, the number for SS is about twice that for RR. At 120 min, the number for SS is approximately 2.5 times that for RR. At a longer incubation time, analyzing the number is difficult because of the shorter stretching distance of the DNA. In conclusion, the number of micro-loops per 100 DNA molecules for SS is always larger than those for RR. In other words, the probability of forming micro-loops for SS is higher than that for RR, which is consistent with the results obtained from the experiments by using magnetic tweezers.

### Statistics of the Volume of Completely Condensed DNA

**Figure 10:**
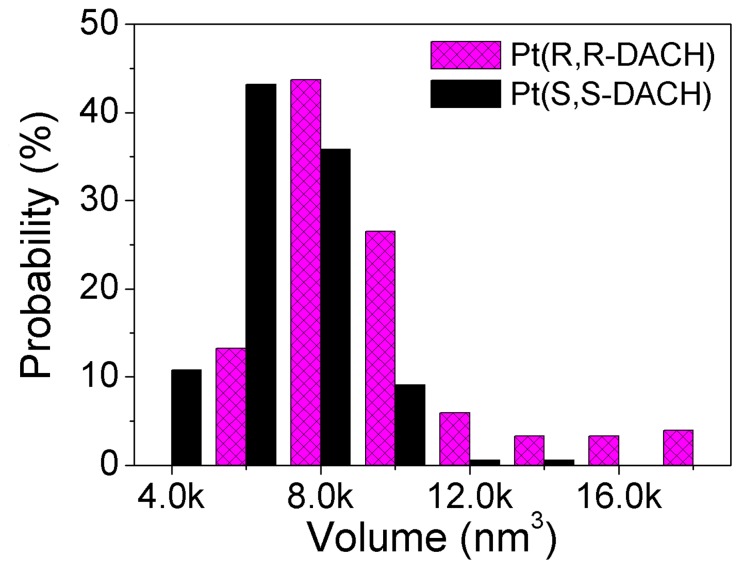
Probability distributions of the volumes of completely condensed DNA molecules. Approximately 150 DNA molecules were analyzed for each group of data. The completely condensed DNA molecules were obtained by incubating with 600 µM Pt(R,R-DACH) or Pt(S,S-DACH) for 16 h.

According to the statistical results of the average stretching distance of DNA molecules incubated with 600 µM Pt-DACH for 16 h, the DNA condensation degree for SS is higher than that for RR. However, because almost all DNA molecules are condensed into 3D compact hemispherical structures, it is more accurate to measure the DNA condensation degree by comparing the volume of completely condensed DNA molecules. The volume is approximately calculated by multiplying the area of DNA on mica surface by the height of DNA. The probability distributions of the volumes are shown in Figure 10. From statistical analyses of about 150 DNA molecules (the completely condensed DNA is difficult to absorb on the mica probably because of its small volume, which means that the sample size is small), the average DNA volume is 9304.6±455.1 nm^3^ (mean ±95% confidence interval) for RR and is 6975.9±238.1 nm^3^ for SS (the values are relevant in a relative sense because the height of DNA in AFM images are influenced by several factors). The volume of completely condensed DNA for RR is approximately 1.3 times that for SS. In other words, the DNA condensation degree for SS is higher than that for RR at the same concentration, which is consistent with the results obtained from the experiments by using magnetic tweezers.

### Measurement of the Amount of Platinum Complexes Bound to DNA by ICP-MS

**Figure 11:**
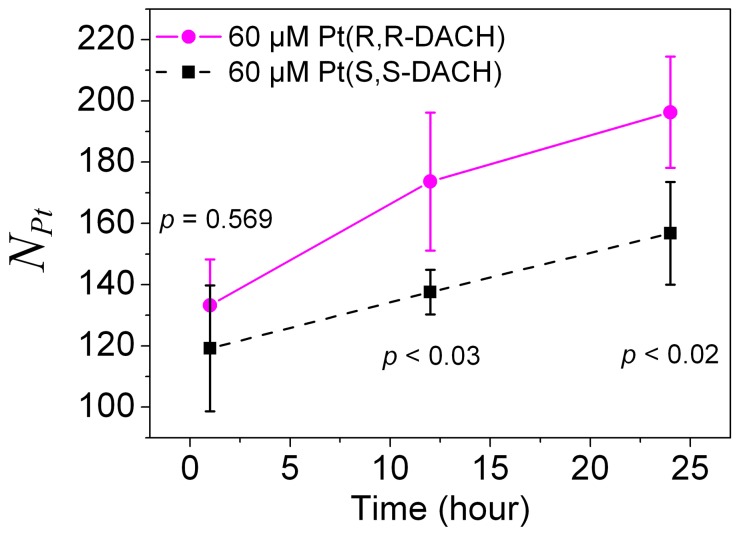
Numbers of Pt-DACH molecules per one DNA molecule (*N_Pt_*). The concentration of Pt-DACH was 60 µM. Each data point was the mean of four independent measurements. The errors corresponded to 95% confidence intervals. The difference was considered statistically significant when the *p* value of two-sample t-test was less than 0.05.

The amounts of Pt-DACH bound to DNA are measured by the inductively coupled plasma mass spectrometry (ICP-MS) and the numbers of Pt-DACH molecules per one DNA molecule are given (Figure 11). The concentration of Pt-DACH is 60 µM. The data of 600 µM is not given because DNA molecules incubated with 600 µM Pt-DACH are condensed into compact hemispherical structures with dimensions of dozens of nanometers, which are difficult to bind to the QIAquick column. The Pt-DACH molecules that are counted contain not only those which form bifunctional adducts, such as diadducts, micro-loops and long-rang cross-links, but also those form monofunctional adducts, namely, monoadducts. The difference between RR and SS groups is tested using the two-sample t-test and is considered statistically significant when the *p* value is less than 0.05. As can be seen in Figure 11, although the concentration of Pt-DACH is relatively high (*r_i_* is about 3), not all purines in DNA are bound by Pt-DACH. At 1 h, the number for RR is almost the same as that for SS. About 125 Pt-DACH molecules are bound to one DNA molecule. However, at both 12 h and 24 h, the number for RR is about 1.25 times that for SS, which indirectly proves that the higher proportions of micro-loops for SS compared with those for RR are responsible for the higher DNA condensation degree.

### Proposed Model

**Figure 12:**
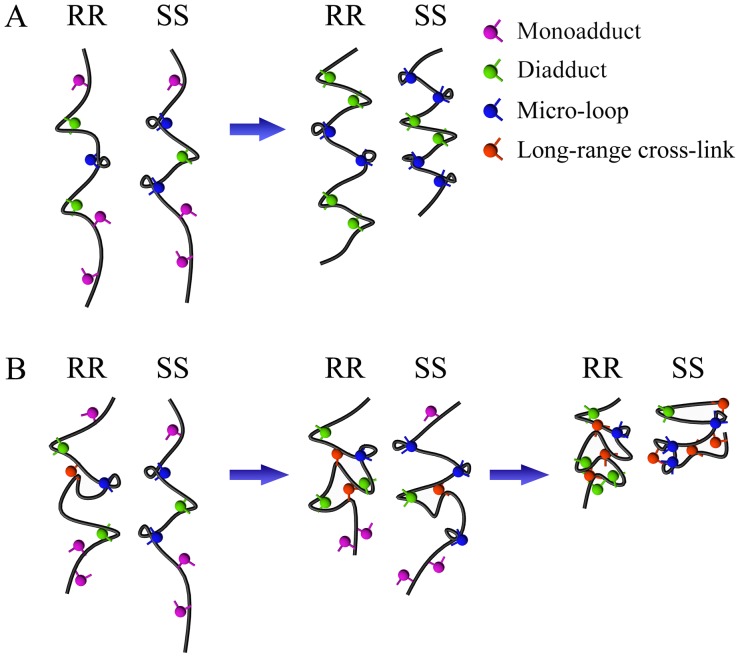
Proposed model for DNA condensation induced by Pt-DACH at low (A) and high concentrations (B).

Based on the results, we propose a model to explain the differences between RR and SS in terms of DNA condensation (Figure 12). RR and SS form monoadducts and diadducts with purines in DNA molecules. However, the diadduct formation rate of SS is smaller than that of RR. Thus, there are more opportunities to form micro-loops or long-range cross-links for SS compared with those for RR. Two reactions, namely, the formations of diadducts and micro-loops, occur in the process of DNA condensation induced by Pt-DACH at low concentrations (Figure 12A). Although the diadduct formation rate of RR is larger than that of SS, the difference between RR and SS in terms of the average DNA condensation rate is not significant in the initial phase because of the greater contribution of micro-loops to DNA condensation and the higher probability to form micro-loops for SS compared with that for RR. The diadduct formation rate decreases rapidly over time, whereas the formation rate of micro-loops decreases relatively slowly. Thus, the average DNA condensation rate for SS is higher than that for RR in the later phase. The difference between RR and SS in terms of DNA extension increases with time. Three reactions, namely, the formations of diadducts, micro-loops, and long-range cross-links, occur in the process of DNA condensation induced by Pt-DACH at high concentrations (Figure 12B). Initially, all these three reactions occur for RR. Nevertheless, only the formations of diadducts and micro-loops occur for SS. The average DNA condensation rate for RR is higher than that for SS because of the combined effect of the high formation rates of diadducts and long-range cross-links of RR. In the middle phase, the instantaneous DNA condensation rate for SS increases with the advent of long-range cross-links. However, the average DNA condensation rate is still lower than that for RR. In the final phase, the DNA condensation degree for SS is greater than that for RR because more micro-loops and long-range cross-links are formed for SS. In short, the major differences in interaction with DNA are the higher diadduct formation rate and the smaller proportions of micro-loops and long-range cross-links for RR compared with those for SS.

The formation of hydrogen bond may be responsible for the higher diadduct formation rate of RR compared with that of SS. Since 1,2-GG adduct makes up the majority of all adducts (60%–65%), it will be discussed in detail below. Diaquated Pt-DACH binds to DNA in a two-step process with two adjacent guanines as the preferred binding sites. First, Pt-DACH rapidly forms monoadduct at 3′-G [29], and then binds to the other 5′-G. X-ray crystal structure [41] indicated the presence of a hydrogen bond between the pseudoequatorial NH hydrogen atom of the RR ligand and the O6 atom of the 3′-G, which is consistent with deduction of the molecular model [22], [27]. The molecular model also revealed the possible presence of a hydrogen bond between the pseudoequatorial NH hydrogen atom of the SS ligand and the O6 atom of the 5′-G. The difference between the two hydrogen bonds is the position of O6 atom of G, which is the result of the chiral carbon atom of the chelate ring. The function of the additional hydrogen bond is very important both in the kinetics of the binding process and in the stabilization of the adduct structure [29]. Combined with the binding process, we can speculate that after the first binding step at 3′-G, the formation of hydrogen bond between RR and the O6 atom of the 3′-G will stabilize the position of RR and accelerate the second binding step. The second binding step will take a long time for SS because no hydrogen bond forms between SS and the O6 atom of the 3′-G. Therefore, the diadduct formation rate of RR is higher than that of SS. Our experiment in vitro excludes the differences between RR and SS in terms of platinum accumulation and interaction with proteins in vivo and gives a more realistic difference in DNA binding between the two enantiomers.

Still, there are issues that remain to resolve. For example, whether the different diadduct formation rates are related to the different antitumor activities between RR and SS; whether micro-loops or long-range cross-links are formed in cells, or are related to the mutagenicity of Pt-DACH.

### Conclusions

In this paper, the mechanical properties of λ-DNA incubated with RR or SS were studied using magnetic tweezers. Both RR and SS coordinate to DNA and decrease the persistence length from approximately 45 nm to approximately 15 nm. However, the effect of RR on DNA persistence length is greater than that of SS, which can be attributed to the higher diadduct formation rate of RR compared with that of SS.

DNA condensation induced by RR or SS of different concentrations was also studied by using both magnetic tweezers and AFM. When DNA molecules are incubated with Pt-DACH of low concentrations, from the magnetic tweezers experiments we find that the curves of DNA extension versus time can be fitted well by a double-exponential decay model, which are assumed to correspond to two concurrent pseudo-first order reactions. The rates of reactions for RR are different from those for SS. Correspondingly, under the same low concentration of Pt-DACH, the AFM images show that only local DNA bending, which is mainly the result of the formation of diadducts, and micro-loops are formed. The probability of forming micro-loops for SS is higher than that for RR. When DNA molecules are incubated with SS of high concentrations, the magnetic tweezers data show that the initial phases of the curves of DNA extension versus time can be fitted by a double-exponential decay model and the last phases of the curves can be fitted by a single-exponential decay model, implying that three pseudo-first order reactions occur. However, the start time of one reaction of them lags the start time of the other two reactions. Although the curves for RR can be fitted by a single-exponential decay model, obviously more than one reaction occurs, as inferred from the discontinuous change in the slope of the instantaneous rate versus time curve. Correspondingly, under the same high concentration of Pt-DACH, the AFM images show that the local DNA bending, micro-loops and long-range cross-links are formed. The start time of long-range cross-links for SS lags that for RR. Both magnetic tweezers and AFM data show a common feature that the final DNA condensation degree for SS is higher than that for RR under any concentration of Pt-DACH. Based on the hypothesis that the process of DNA condensation in the magnetic tweezers experiment is similar to that in the AFM experiment, we propose a model to explain the process of DNA condensation induced by RR or SS. The differences in terms of interaction with DNA are attributed to the higher diadduct formation rate and the smaller proportions of micro-loops and long-range cross-links for RR compared with those for SS.

The present study facilitates the understanding of the effect of chirality of Pt-DACH on its interaction with DNA and the kinetics of DNA condensation induced by platinum complexes. The importance of the chirality of drugs is proven. The magnetic tweezers experiment is proved to be a simple but powerful method in measuring the kinetics of platinum complexes binding to DNA.

## Materials and Methods

### Chemicals and Reagents

[Pt(R,R-DACH)](NO_3_)_2_ and [Pt(S,S-DACH)](NO_3_)_2_, which quickly formed positively charged monoaquated and diaquated species once dissolved in water, were obtained from the Institute of Precious Metal in Kunming, China. The stock solutions of Pt-DACH (10 mM) were stored in the dark at 4°C for one month. The working solutions were diluted by HEPES buffer (10 mM 4-(2-Hydroxyethyl) piperazine-1-ethanesulfonic acid (HEPES, Sigma-Aldrich), 30 mM CH_3_COONa (Sigma-Aldrich), pH 7.4). Bovine serum albumin (BSA), Pluronic F-127 surfactant, and Thiourea were obtained from Sigma-Aldrich. Anti-digoxigenin was obtained from Roche. All solutions were made with 18.2 MΩ deionized water purified by the Milli-Q Water Purification System (Millipore Corporation, France). All other chemicals were analytical grade reagents.

### Magnetic Tweezers Apparatus

The magnetic tweezers setup was purchased from Pico Twist Company (France). In brief, it was made up of an inverted microscope objective, a microfluidics flow cell and a pair of permanent magnets. Under the flow cell, the microscope objective (Olympus 100×, numerical aperture [NA] = 1.2, oil immersion) was used to observe the beads in real time. In the flow cell, the DNA molecule was bound to the bottom of the cell at one end. The other end was tethered to a super-paramagnetic bead (MyOne, Dynabeads, Invitrogen). Above the flow cell, there was a set of magnets producing a strong field gradient to exert a force on beads. The force was varied by changing the position of the magnets relative to the beads. The beads can be rotated through rotating the magnets. The detailed procedure of the flow cell assembly and the standard operation of the magnetic tweezers were described by Croquette and other researchers [48], [49].

### λ-DNA Preparation for Magnetic Tweezers Study

The bacteriophage λ-DNA (New England Biolabs), which has two 12-nt cohesive termini, was separately annealed with two 12-nt labeled oligomers (labeled by biotin and digoxigenin, respectively). These oligomers have complementary sequences to the overhangs. The 12-nt oligomers were obtained from Sangon Biotech (Shanghai). The details of the DNA construction were described by Lehner [50].

### Single Molecule Measurement by Magnetic Tweezers

The force-extension curves of DNA incubated with Pt-DACH were measured to investigate the effects of Pt-DACH on DNA elasticity. RR or SS at a concentration of 60 µM was injected into the flow cell and incubated with DNA while keeping DNA stretched by a large constant force (approximately 6 pN). The stretching force prevented the formation of micro-loops and long-range cross-links. At a given time, the force-extension curve was recorded and fitted by the WLC model [51], [52] to obtain the persistence length at that time. The persistence length is a parameter to describe the elasticity of the double helix [53].

The extensions of DNA incubated with Pt-DACH at different concentrations were measured with time. RR or SS at a given concentration was injected into the flow cell while keeping DNA stretched by a large force (approximately 4 pN). The stretching force was then reduced to approximately 0.8 pN and the DNA extension was recorded immediately.

### 2-kb DNA Preparation for AFM Study

The 2-kb DNA fragment was generated by PCR using pBR322 plasmid DNA (New England Biolabs) as a template [54].

### AFM Sample Preparation and Imaging

The DNA molecules incubated with Pt-DACH were scanned using AFM to study the effects of Pt-DACH on DNA conformation. 2-kb DNA at a concentration of 6.1 ng/µL was incubated with RR or SS in HEPES buffer at 37°C in the dark. The concentration of Pt-DACH was 60 µM or 600 µM (the molar ratio of Pt-DACH complex to nucleotide, *r*_i_, was approximately 3 or 30). At specific time intervals, an aliquot of the reaction mixture was extracted and diluted to a DNA concentration of 0.61 ng/µL for AFM scanning.

The scanning was performed in air with a multi-mode AFM with a nanoscope IIIa controller (Digital Instruments, Santa Barbara, CA, USA) in the tapping-mode. The silicon probe RTESP14 from Veeco (America) with a resonance frequency of 314–316 kHz was employed. ‘E’ scanner was used. The scan frequency was 1 Hz per line, and the scan size was from 1 µm×1 µm to 5 µm×5 µm. Image analysis was performed using the Nanoscope III 5.31r1 software package. DNA measurement was semi-automatically done using the Image J software.

### ICP-MS Sample Preparation and Measurement

2-kb DNA at a concentration of 6.1 ng/µL was incubated with RR or SS in HEPES buffer at 37°C in the dark. The amount of DNA was 800 ng. The concentration of Pt-DACH was 60 µM (*r*_i_ was approximately 3). At specific time, the binding reaction of Pt-DACH to DNA was stopped by the addition of 10 mM thiourea for 10 min at room temperature without displacing Pt-DACH from the DNA [55]. Then the sample was purified using a QIAquick PCR purification Kit to remove free Pt-DACH and thiourea, where the washing step was repeated three times. After wet-digestion, the amount of Pt of the sample was measured by ICP-MS (ELAN DRC II, PerkinElmer, USA) [56]. The detection limit was 0.01 ng/mL Pt.

### Formulas

The force-extension curve of a single DNA molecule was fitted by the formula [57],
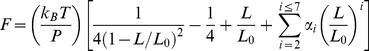
(1)with α_2_ = −0.5164228, α_3_ = −2.737418, α_4_ = −16.07497, α_5_ = −38.87607, α_6_ = 39.49944, and α_7_ = −14.17718, where *F* is the stretching force, *L* is the DNA extension, *k_B_* is the Boltzmann's constant, *T* is the absolute temperature, *P* is the persistence length and *L*_0_ is the contour length of DNA molecule.

The single-exponential function used to fit the DNA extension versus time curves was
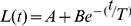
(2)where *L*(*t*) is the DNA extension at time *t,* and *T* is the time constant. The double-exponential function was
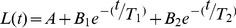
(3)where T1 and T2 are time constants.
